# Raft Selectivity of a Cholesterol Probe Capable of Forming an H-Bond with Phospholipids

**DOI:** 10.3390/molecules31132297

**Published:** 2026-07-01

**Authors:** Ivan Ryzhov, Eugenia Rapoport, Polina Obukhova, Alexander Tuzikov, Mariia Sokolova, Darya Anisimova, Oxana Galanina, Sergey Khaidukov, Stephen Henry, Nicolai Bovin

**Affiliations:** 1 Shemyakin-Ovchinnikov Institute of Bioorganic Chemistry, Russian Academy of Sciences, 117997 Moscow, Russia; imryzhov@gmail.com (I.R.); eugenia_rapoport@mail.ru (E.R.); anruma@yandex.ru (P.O.); alextuzikov@yandex.ru (A.T.); sms21297@gmail.com (M.S.); d.petrakova2000@yandex.ru (D.A.); galox@inbox.ru (O.G.); khsergey54@mail.ru (S.K.); 2 National Medical Research Center for Obstetrics, Gynecology and Perinatology Named After V. I. Kulakov, 117198 Moscow, Russia; 3 School of Engineering, Auckland University of Technology, Auckland 1010, New Zealand; shenry@kodebiotech.com

**Keywords:** cell rafts, cholesterol, glycosphingolipids, phospholipids, FSL, synthetic glycolipids

## Abstract

Selective insertion of lipid probes from the external milieu into raft regions of the cell membrane would enable targeted studies of raft molecular organization and of raft-associated peptide and glycan components. In this work, we compared the insertion of several synthetic glycolipids into raft and non-raft membrane areas of Raji and EA.hy 926 endothelial cells. A glyco-cholesterol derivative in which the 3β oxygen atom is replaced by NH was selected as a candidate raft-selective probe. Although this glycolipid also inserted into other membrane areas, its raft-to-non-raft distribution ratio was higher than that of its *O*-analog (natural 3β-*O*-cholesterol).

## 1. Introduction

Lipid rafts are discrete membrane microdomains [1] where signaling molecules and their hetero-complexes are concentrated, allowing protein and glycosphingolipid [2] receptors to interact more closely and facilitating the interactions necessary for signal transduction [3]. Selective modification of rafts with regulatory molecules is a promising strategy for influencing intracellular processes, potentially including therapeutic processes. Targeted incorporation of molecules into lipid rafts is also a possible approach to selective raft labeling, a task that still lacks reliable and universal solutions [4].

Recently, we developed an approach for the modification of cell membranes with almost any molecular fragment (glycan, protein, fluorophore, ligands for covalent and non-covalent conjugation, etc. [5,6,7,8,9,10]) based on synthetic functionalized lipids, so-called FSLs (Function-Spacer-Lipid constructs) [10]. These molecules can insert into the cell membrane from extracellular media [11]. From our perspective, this methodology is suitable for creating lipidated probes targeted for selective insertion into raft zones of the membrane. It is also apparent that selecting the right lipid component for such probes is the primary objective, as the lipid component is responsible for anchoring the molecule in the membrane. Analysis of current literature data on the lipid composition of rafts and probes for raft labeling, together with comparison of these data with our set of lipid building blocks for FSL synthesis, led to the following conclusions. Phospholipids with unsaturated acyl chains, such as DOPE, the main lipid block used in FSLs, are not suitable for this task because they are known to aggregate in the L_d_ (liquid-disordered, non-raft) phase of the membrane [12]. The situation with derivatives of phospholipids with saturated acyl chains (for example, DSPE [5]) is less certain: their distribution between L_o_ and L_d_ phases was shown to depend on the lipid composition of the artificial membrane [4,12,13,14], as well as on the nature of the phospholipid head group [4,15]. However, the main argument against the use of phospholipids with saturated acyl chains is that their insertion into living cells is much worse than in artificial membranes [16]. Cholesterol derivatives seemed to be the most suitable option for use as the lipid component of raft-selective probes. Several cholesterol-based fluorescent probes, in which the fluorophore is attached to a side alkyl chain of cholesterol and the hydroxyl group remains intact, are known to partition mainly to the L_o_ phase [4]. In our case, taking into account the orientation of cholesterol in the membrane and the position of the functional part outside the membrane, attaching the functional group to the side chain of cholesterol is counterproductive, and the polar head located close to the membrane surface is the best position for conjugation. On the other hand, we have previously shown that glycolipids with a cholesterol moiety attached via its OH group do not integrate into cholesterol-rich rafts [17]. This is due to replacement of the hydrogen atom at the 3β-hydroxyl of cholesterol. Molecular dynamics simulations demonstrated that this hydrogen atom is required for the formation of *H*-bonds with the polar heads of membrane phospholipids, which is crucial for correct insertion of the cholesterol residue into the tightly packed and organized raft [18]. To solve this contradiction and retain both the possibility of conjugation at the 3β-position of cholesterol and the presence of a hydrogen atom in this position to form *H*-bonds, replacement of the OH group with its isostere, an NH_2_ group, was proposed earlier by Hussey et al. [19,20,21]. We found this approach promising for creating raft-targeted FSLs and synthesized a glycolipid in which blood group A tetrasaccharide was attached to 3β-aminocholesterol via alkylation of the amine [22]. The objective of this study was to evaluate a derivative—in which the NH group of 3β-aminocholesterol retains the capacity to form hydrogen bonds—as a raft-selective probe; specifically, to assess its lateral distribution in the plasma membrane, compared with similar probes lacking the ability to form *H*-bonds via the 3β group.

## 2. Results

### 2.1. Synthetic Glycolipids Used in This Study

Five FSL constructs containing blood group A (type 2) tetrasaccharide (A2) as the functional part (F), and detectable using monoclonal anti-A antibodies, were used in this study; their structures are shown in Table 1. The A2-Ad-*N*Chol construct (**1**) was the primary target of our study because its glycan part is attached through a spacer to 3β-aminocholesterol, which is capable of *H*-bonding with polar heads of lipids in the membrane. Synthesis of A2-Ad-*N*Chol is described in [22], and its NMR and HRMS data can be found in Supplementary Materials, Figures S1–S3. The most suitable comparison for A2-Ad-*N*Chol (**1**) was A2-Av-Chol (**2**), in which the glycan part is attached through a spacer to cholesterol at its 3β-position via oxygen, and the linkers are almost identical, thus A2-Av-Chol (**2**) was used as the primary control for A2-Ad-*N*Chol (**1**) to confirm that replacement of the O atom with an NH group in the cholesterol anchor is the key factor affecting membrane-domain distribution of the constructs. The following two additional glycolipids with an *O*-cholesterol lipid and different spacer-arm lengths were studied: A2-Chol (**3**), with the shortest spacer (only the aminopropyl linker of the glycan), and A2-CMG_2_-Av-Chol (**4**), with a long CMG-type spacer (built from glycine and carboxymethylglycine (CMG) units), the linearity of which is supported by repulsion of negative charges on carboxyl groups [23]. In addition, A2-Ad-DOPE (**5**) was also used; this differs from (**1**), (**2**), (**3**) and (**4**) in the nature of the lipid tail. Synthesis of glycolipid constructs (**2**–**5**) was described earlier [5,6].

### 2.2. Insertion Efficiency

To evaluate the cytotoxicity and induction of apoptosis by the A2-Ad-*N*Chol construct, after insertion cells were double-stained with 7-AAD and Annexin-V. The percentage of necrotic and apoptotic cells was less than 6% and 12%, respectively (Supplementary Materials, Figure S4). Thus, after insertion, A2-Ad-*N*Chol did not affect cell viability and did not induce apoptosis.

**Figure 1:**
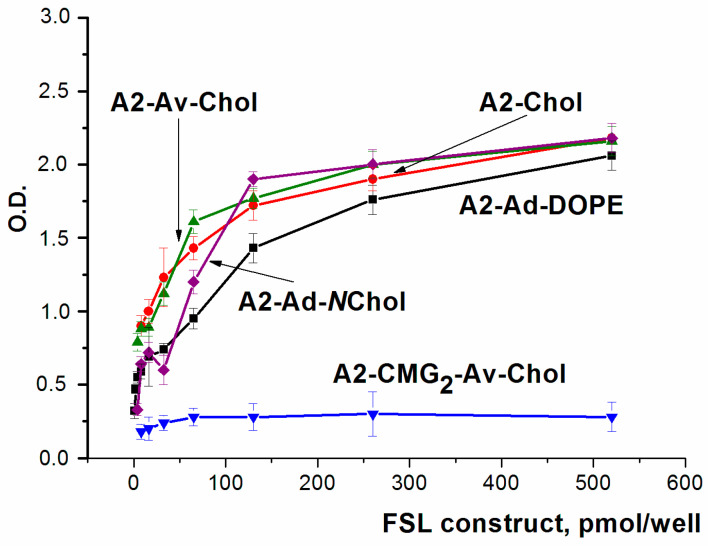
Binding of anti-A monoclonal antibody to FSLs inserted into a PC-Chol layer (2.5 µg/mL PC, 5 µg/mL Chol, 1 h), EIA data. Results shown include data from four experiments; error bars represent the standard deviation.

**Figure 2:**
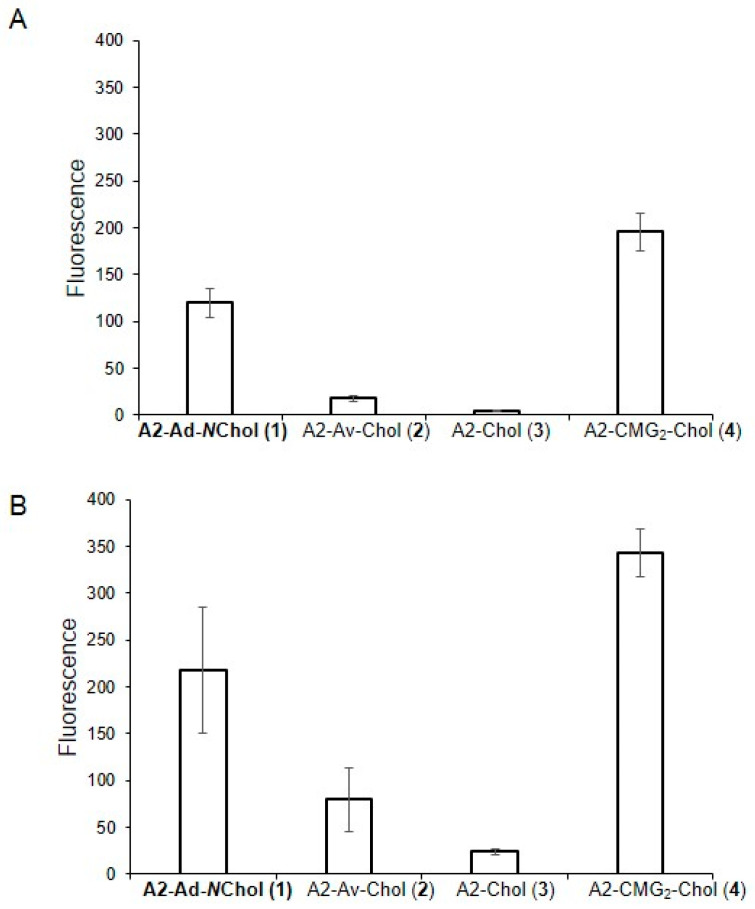
Insertion of FSLs into EA.hy 926 cells (**A**) and Raji cells (**B**). The amount of inserted FSL was detected with anti-A antibodies followed by staining with FITC-labeled secondary antibodies. Flow cytometry data are shown; the y-axis shows fluorescence increase, calculated as [(F_i_/F_0_) × 100] − 100, where F_i_ is the geometric mean fluorescence intensity of cells after insertion of FSL and incubation with anti-A and secondary antibodies, and F_0_ is the geometric mean fluorescence intensity of cells stained only with anti-A and secondary antibodies. Results shown include data from three experiments, and error bars represent the standard deviation.

The ability of the A2-Ad-*N*Chol (**1**) construct to integrate into membranes, compared with other FSLs (**2**–**5**) lacking the key structural motif R-*N*Chol (Table 1), was studied in the following two test systems: an artificial PC-Chol layer formed on polystyrene (Figure 1) and a cell membrane (EA.hy 926 and Raji cells, Figure 2). In all analytical systems used, insertion was performed by 1–1.5 h incubation of the FSL solution with the artificial PC-Chol layer or cell membrane under conditions previously found to be optimal [16], except for A2-Ad-*N*Chol, which is novel. The FSL concentrations used were in the lower micromolar range, well below their CMCs (which are in the lower millimolar range); therefore, FSLs insert into the membrane directly from solution rather than from micelles. Insertion was assessed using anti-A antibodies.

The concentration-dependent insertion of FSLs (**1**–**5**) into the PC-Chol layer formed on polystyrene, as assessed by EIA, is shown in Figure 1. The amino-cholesterol construct A2-Ad-*N*Chol is best compared with the normal cholesterol construct A2-Av-Chol, which is structurally almost identical except for the absence of the NH group capable of acting as a *H*-bond donor. The concentration dependences of these two glycolipids were close to each other. Comparison with other FSLs showed that the insertion level of the A2-Chol construct was almost the same, whereas the DOPE construct inserted slightly less efficiently. The insertion level of A2-CMG_2_-Av-Chol was significantly lower than for all other constructs.

The insertion of FSLs into the plasma membrane of EA.hy 926 (endothelial) and Raji (lymphoblast-like) cells was studied by flow cytometry; the results are shown in Figure 2A,B. The amino-cholesterol construct A2-Ad-*N*Chol inserted into EA.hy 926 and Raji cells better than its *O*-analog, A2-Av-Chol (Figure 2A,B), and also better than A2-Chol, but less efficiently than A2-CMG_2_-Av-Chol, as evidenced by the fluorescent signal after antibody binding. The fluorescence values for EA.hy 926 cells were 120 (A2-Ad-*N*Chol), 18 (A2-Av-Chol), five (A2-Chol), and 196 (A2-CMG_2_-Av-Chol); for Raji cells, they were 218 (A2-Ad-*N*Chol), 84 (A2-Av-Chol), 24 (A2-Chol), and 343 (A2-CMG_2_-Av-Chol). The fact that the latter inserts into artificial lipid layer less efficiently than the others, but into cells more efficiently than the others, is consistent with our previous results [16]. The insertion profiles of the probes into EA.hy 926 and Raji cells were nearly identical, although the degree of insertion was approximately twofold higher in the latter.

### 2.3. Site-Selectivity of A2-Ad-NChol Insertion into Rafts

**Figure 3:**
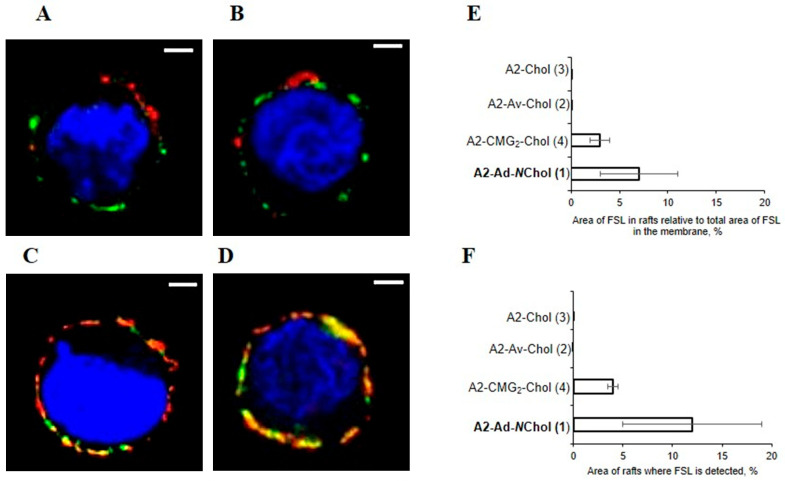
Localization of FSLs inserted into EA.hy 926 cells, with confocal microscopy data quantified using ImageJ [25]. (**A**), A2-Chol. (**B**), A2-Av-Chol. (**C**), A2-CMG_2_-Av-Chol. (**D**), A2-Ad-*N*Chol. FSLs were incubated with EA.hy 926 cells for 1 h at 5 µm. Rafts stain green with cholera toxin B conjugate, FSLs stain red with immunofluorescence, and nuclei stain blue with DAPI. Inset white scale bar is 5 µm. (**E**), FSL content in rafts (yellow), expressed as % of the red + yellow (total inserted FSL) staining. (**F**), area of rafts with FSL entrapped relative to total area of rafts, %. Results shown include data from four independent experiments; data were averaged for 12 cells in each case.

**Figure 4:**
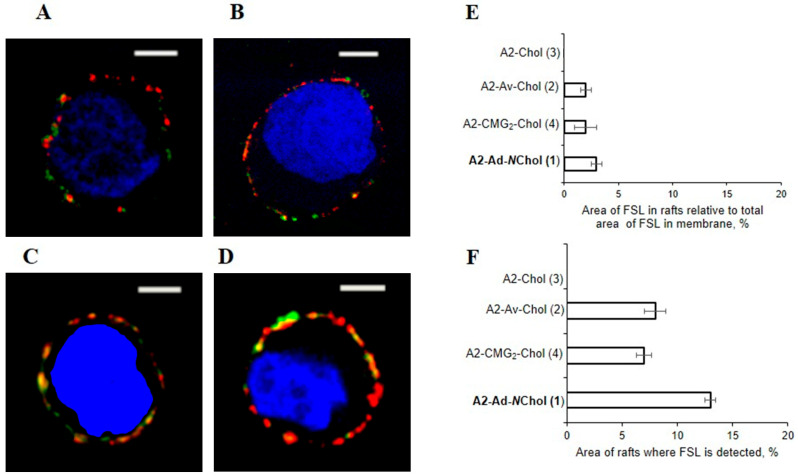
Localization of FSLs inserted into Raji cells, with confocal microscopy data quantified using ImageJ [25]. (**A**), A2-Chol. (**B**), A2-Av-Chol. (**C**), A2-CMG_2_-Av-Chol. (**D**), A2-Ad-*N*Chol. FSLs were incubated with Raji cells for 1 h at 5 µm. Rafts stain green with cholera toxin B conjugate, FSLs stain red with immunofluorescence, and nuclei stain blue with DAPI. Inset white scale bar is 5 µm. (**E**), FSL content in rafts (yellow), expressed as % of the red + yellow (total inserted FSL) staining. (**F**), area of rafts with FSL entrapped relative to total area of rafts, %. Results shown include data from four independent experiments; data were averaged for 8 cells in each case.

The lateral distribution of FSLs inserted into the plasma membrane of EA.hy 926 cells was assessed by confocal microscopy. Rafts were stained with cholera toxin B [24], which binds to endogenous GM1 ganglioside found predominantly in rafts, and FSL constructs were stained with anti-A2 antibody. Insertion of FSLs into rafts can be determined from merged staining images (Figure 3A–D), and their relative percentages were determined with ImageJ (Figure 3E). The proportion of A2-Ad-*N*Chol detected in rafts was higher than that of A2-CMG_2_-Av-Chol, whereas the other two cholesterol-containing FSLs were not detected in rafts. When the proportion of rafts containing A2-Ad-*N*Chol to total area of rafts was considered, the value increased (Figure 3F). In Raji cells, three FSL constructs, A2-Av-Chol, A2-CMG_2_-Av-Chol and A2-Ad-*N*Chol, were localized in rafts (Figure 4E) in approximately the same quantities, although the proportion of rafts containing A2-Ad-*N*Chol to total area of rafts was higher (Figure 4F).

The above results were obtained under conditions that are not necessarily optimal for segregation of FSLs between raft and non-raft zones. In particular, after 1 h incubation, FSL insertion occurs to a degree sufficient for further studies (confocal microscopy, isolation of FSL-containing microvesicles derived from the modified cell, etc.), but one hour may not be sufficient to achieve equilibrium in the distribution of FSLs between membrane zones. In addition, it is possible that the FSL concentration of 5 µM during insertion may be too high, while at a lower concentration the distribution could be more selective. To evaluate this, we compared the insertion of A2-Ad-*N*Chol and A2-Av-Chol at concentrations of 1 vs 5 µM, as well as insertion for 5 h vs 1 h (at both concentrations). Variation in concentration did not influence the localization of the inserted constructs. After prolonged incubation (5 h), the distribution of A2-Ad-*N*Chol between raft and non-raft zones was almost the same as after 1 h incubation. A2-Av-Chol was found in both raft and non-raft areas (unlike the image after 1 h incubation, when it was absent in rafts, Figure 3), although the percentage of raft area with inserted construct was lower for A2-Av-Chol (10%) than for A2-Ad-*N*Chol (15%) (Supplementary Materials, Figure S5).

## 3. Discussion

The idea of replacing the 3β-OH group in cholesterol with an NH_2_ group, followed by alkylation of the amino group to conjugate a functional group to 3β-aminocholesterol, was originally proposed in [20,21,26]. To demonstrate the applicability of this approach, an Alexa-containing analog of cholesterol (*N*Chol), labeled by the amino group, was inserted into Jurkat cells at a concentration of 10 µM for 1 h, under conditions close to those used here, and the rafts were similarly stained with cholera toxin B [21]. Although the co-localization percentage of *N*Chol within rafts was not quantified by the authors, the published confocal image appears to show significant raft labeling. In addition, the lateral distribution of several labeled *N*Chol derivatives across the membrane of Jurkat cells was studied; however, no clear conclusions about their co-localization within rafts were drawn [26]. In [20], under the same conditions, the confocal image can be interpreted as co-localization over almost the entire membrane. To interpret the results presented in the cited publications, it is necessary to understand that, in Jurkat cells, rafts are small and cholera toxin visualizes them over the entire membrane [27], not in spots as is usually the case. Therefore, it is not possible to draw conclusions from the cited works about the preference of the *N*Chol probe for integration into rafts. However, we found this approach promising for the design of probes for selective insertion into rafts and aimed to study in detail the localization of *N*Chol probes inserted into the cell membrane. In the cells used in this study, the proportion of rafts is no more than 10% of the membrane area, so we were able to distinguish raft and non-raft areas and quantify the partitioning of the glycolipid constructs between them. It should also be noted that, in the above-cited publications, the *N*Chol derivatives had hydrophobic “heads”, in contrast to our work, where the “head” is a hydrophilic tetrasaccharide.

**Figure 5:**
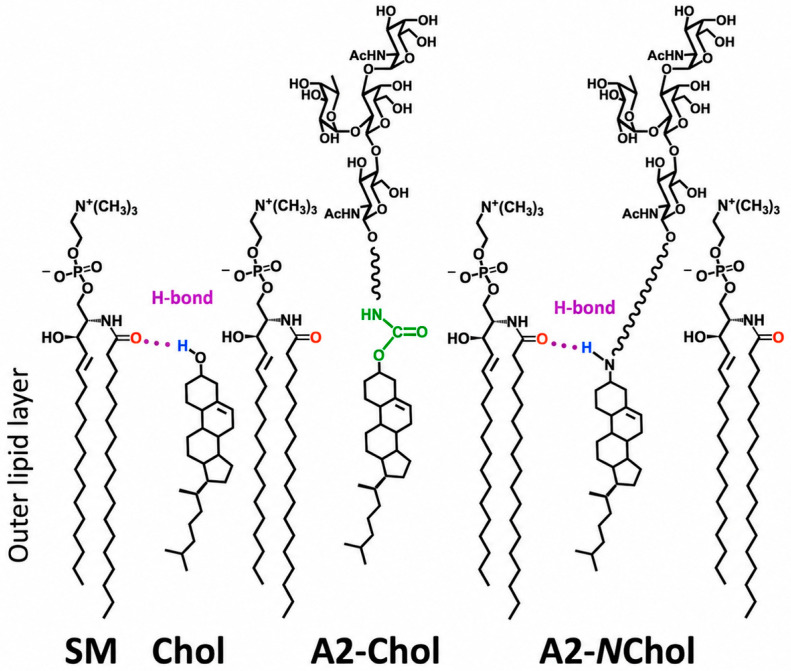
Schematic illustration of the difference between insertion of cholesterol (Chol), *N*Chol, and *O*Chol derivatives into a membrane raft. Sphingomyelin (SM) is chosen as a typical component of the raft membrane. Formation of an *H*-bond between the proton of the 3β-substituent of sterol (blue) and the oxygen of the amide moiety in SM (red) is shown in purple, carbamate moiety of *O*Chol derivative unable to form analogous *H*-bond is shown in green.

The selectivity of aminocholesterol-construct insertion into membrane rafts depends on a feature of the 3β-substituent of the sterol moiety upon insertion into a tightly packed membrane raft. This mechanism is illustrated in Figure 5. Within lipid rafts, cholesterol molecules adopt a “vertical” orientation, with their hydroxyl group positioned near the outer membrane surface. The ability of cholesterol to stabilize the L_o_ phase results from several factors, including hydrogen bonding between the proton of the hydroxyl group and polar atoms (oxygen or nitrogen) located in the polar head groups of other raft components (for example, the amide function of sphingomyelin, as shown in Figure 5). When cholesterol-containing glycolipids are synthesized by substituting the proton of the 3β-OH group with another functional moiety, the resulting construct loses its ability to act as a hydrogen-bond donor in the outer membrane layer. This prevents proper incorporation into the raft with its highly ordered and densely packed lipid organization.

Substitution of the hydroxyl group with an amine can act as an effective isosteric replacement that preserves hydrogen-bond donor capacity. 3β-Aminocholesterol derivatives obtained via *N*-alkylation retain one N-H bond and thus remain capable of engaging in interfacial hydrogen bonding, thereby facilitating their incorporation into membrane rafts. Nevertheless, aminocholesterol derivatives retain their ability to incorporate into non-raft membrane domains, which is relevant for the interpretation of the results discussed below. It should also be noted that the corresponding cholesterol amides obtained via acylation of 3β-aminocholesterol exhibit reduced raft affinity due to decreased hydrogen-bond donor strength [21].

The data obtained in our study are generally consistent with the proposed mechanism. First, we assessed the overall ability of cholesterol glycolipids to incorporate into the cell membrane, irrespective of their localization within specific domains. The aminocholesterol derivative (A2-Ad-*N*Chol) exhibited a higher level of insertion than its *O*-cholesterol analog (A2-Av-Chol) in both EA.hy 926 and Raji cells. Thus, substitution of an oxygen atom with an NH group appears to be a factor that facilitates insertion into raft membranes, although it does not render the compound raft-specific. Unexpectedly, the compound A2-CMG_2_-Av-Chol demonstrated the best overall insertion characteristics; the observed selectivity remains unexplained yet holds promise for enhancing the tropism of probes toward rafts in future studies.

The insertion extent of A2-Ad-*N*Chol into the artificial PC-Chol layer on polystyrene surface was almost indistinguishable from that of the analogous probe with *O*-substituted cholesterol. The artificial PC-Chol layer represents a highly simplified lipid-coated environment and lacks the structural organization characteristic of lipid rafts. Thus, the ability of the A2-Ad-*N*Chol to insert into this surface suggests that it can also integrate into non-raft membrane regions. In other words, there should be no specific preference for rafts, although the *N*-derivative does have the ability to interact with the raft plasma membrane, which is absent in the *O*-analogs. Indeed, confocal microscopy data from EA.hy 926 cells modified with A2-Ad-*N*Chol indicate its location in both rafts and areas outside rafts. In contrast, other cholesterol FSLs, in which the amino group key for hydrogen bonding is absent, were not detected in rafts (EA.hy 926 cells) or their amount was lower than that of A2-Ad-*N*Chol (Raji cells); that is, the *H*-bond appeared to be a “vector” directing the FSL into rafts. Although the amount of total FSLs inserted into rafts is seemingly low, it should be considered in the context that A2-Ad-*N*Chol retains the ability to insert into non-raft regions, which dominate over raft regions in cells, despite the structural modification. Therefore, a more indicative index of selectivity is the raft area occupied by the *N*Chol probe compared with the total raft area (Figure 3F and Figure 4F), which reached 13%. Most importantly, the construct chosen for comparison with A2-Ad-*N*Chol, namely A2-Av-Chol, was not detected in raft regions (EA.hy 926 cells), or its amount was lower (Raji cells). Prolonged incubation (5 h) of FSLs with EA.hy 926 did not affect construct distribution. Apparently, insertion of the *O*-version initially occurs within the more fluid regions of the membrane—areas that are not only more receptive to exogenous lipids but are also far more widely represented within the cell membrane than rigid raft domains—followed by partial redistribution of the inserted probe via a mechanism that remains unclear.

Thus, substitution of an oxygen atom with a nitrogen atom in the cholesterol structure results in the emergence of tropism (selectivity) of the probe toward raft domains, albeit without ensuring a high degree of raft specificity.

## 4. Materials and Methods

### 4.1. Materials

DMEM-F12, RPMI-1640 media, glutamine, cholera toxin B subunit (CTB) conjugated with Alexa Fluor^®^ 488, anti-mouse IgM, anti-mouse IgG conjugated with Alexa Fluor 594, and 4′,6-diamidino-2-phenylindole (DAPI) were from Invitrogen Co. (Carlsbad, CA, USA). Fetal calf serum (FCS), carbohydrate-free BSA, Tween 20, phosphate-buffered saline, Mowiol^®^ 4-88, and goat anti-mouse IgM and IgG FITC conjugates were from Sigma (St. Louis, MN, USA).

Mouse monoclonal anti-A antibodies (clone A3) were obtained from the National Medical Research Center for Hematology (Moscow, Russia). Horseradish peroxidase-labeled streptavidin (Str-HRPO) and goat anti-mouse Ig labeled with biotin were from SouthernBiotech (Birmingham, AL, USA). MaxiSorp 96-well microtiter immunoplates were obtained from Thermo Fisher Scientific (Rochester, MN, USA). Salts and H_2_O_2_ were analytical grades from Merck (Kenilworth, NJ, USA). Egg phosphatidylcholine was from Lipoids (Heidelberg, Germany), and cholesterol was from Avanti Polar Lipids (Alabaster, AL, USA). All other reagents were from Reachem Corp. (Moscow, Russia).

### 4.2. Cell Culture

Raji cells (human B lymphocytes, ATCC^®^ CCL-86™) were cultured in RPMI-1640 supplemented with 10% FCS and 2 mM glutamine at 37 °C in a humidified atmosphere of 5% CO_2_. EA.hy 926 cells, provided by Dr. C.-J. Edgell (Chapel Hill, NC, USA), are immortalized cells that retain the morphological and functional properties of human umbilical endothelial cells [28]. These cells were cultured in DMEM-F12 with the same additives and under the same conditions.

### 4.3. Insertion of FSL into Cells

Confluent EA.hy 926 cells were washed three times with DMEM-F12 containing 0.3% FCS and incubated with FSL (5 µM) at 37 °C in a humidified atmosphere of 5% CO_2_ for 1 h. A concentration of 0.3% FCS was used because high percentages of serum can interfere with glycolipid insertion into the cell membrane [29]. Versene-detached cells were washed twice with PBS containing 0.2% BSA (PBA) by centrifugation at 90×*g* and 4 °C for 3 min. Anti-A monoclonal antibodies A3 (1:50 in PBA) were added, and cells were incubated for 20 min at 4 °C under agitation, washed, and incubated with FITC-labeled secondary antibodies (1:100 in PBA) for 20 min at 4 °C under the same conditions. Cells were washed with PBA and analyzed by flow cytometry.

Raji cell suspensions were washed with RPMI-1640 containing 0.3% FCS, seeded into a 24-well plate, and incubated with FSL as described above.

The in vitro cytotoxicity assay using Annexin V and 7-AAD (7-aminoactinomycin D) was performed using reagent concentrations recommended in the instructions supplied with the apoptosis kit, at 4 °C for 15 min.

### 4.4. Flow Cytometry

After washing, cells were mixed with 2 mL of PBS. Flow cytometry was performed at room temperature using a FACScan instrument (Becton-Dickinson Co., Franklin Lakes, NJ, USA) equipped with FlowJo V10.5.3 software, or an FC500 cytofluorimeter (Beckman Coulter, Miami, FL, USA) equipped with Kaluza 1.3 software.

### 4.5. Microscopy

FSLs (final concentration, 1 or 5 µM) were added to monolayers of EA.hy 926 cells or suspensions of Raji cells and incubated at 37 °C in a humidified atmosphere of 5% CO_2_ for 1 or 5 h. EA.hy 926 cells were detached with Versene solution, and Raji cells were collected. Cells were washed and incubated with anti-A antibodies (1:100 in PBA) at 4 °C for 20 min and then with anti-mouse IgM conjugated with Alexa Fluor 594 (1:200 in PBA) under the same conditions.

To visualize cell rafts, EA.hy 926 cells were washed with PBS and incubated with cholera toxin B subunit conjugated with Alexa Fluor 488 (1:100 in PBS) on ice for 20 min. A microscope-mounting medium containing 2.4 g of Mowiol 4–88, 6 g of glycerol, 6 mL of water and 12 mL of 0.2 M Tris-HCl (pH 8.5) was placed on a microscope slide, followed by the cell suspension (10 µL). Images were obtained with a Nikon Eclipse TE-2000-E confocal microscope (Nikon, Minato City, Japan) and analyzed with ImageJ (Version G 2.3.0/1.53t/Java1.8.0_172, http://imagej.net/ij/, accessed on 23 June 2026). At least 12 randomly selected cells were analyzed in each experiment.

### 4.6. Solid Phase Assay

Plates were coated with a known excess of lipids, using an ethanolic solution of PC (2.5 µg/mL) and cholesterol (5 µg/mL), calculated to exceed the amount required to cover the plastic surface as described earlier [30]. The plates were incubated at 37 °C for 1 h and then left overnight at 20 °C. FSL constructs (two-fold dilutions starting from 520 pmol per well) in PBS were added, and the plate was incubated for 1 h at 50 °C, washed with PBS containing 0.1% Tween-20 (the washing buffer), blocked with 3% BSA in PBS for 1 h at 37 °C, and washed again. Anti-A monoclonal antibodies A3 (1:200) in PBA were added, and the plates were incubated for 1 h at 37 °C, washed with the washing buffer, incubated with anti-mouse Ig-biotin conjugate (1:2000 in PBA) for 1 h at 37 °C, and washed with the washing buffer. The plate was then incubated with Str-HRPO (1:2000 in PBS/BSA) for 1 h at 37 °C followed by washing. Color was developed by incubation with 0.1 M sodium phosphate/0.1 M citric acid buffer containing 0.04% o-phenylenediamine and 0.03% H_2_O_2_. Absorbance was recorded at 492 nm in a Multiskan MCC/340 plate reader (Perkin Elmer, Turku, Finland). Control wells contained no FSL constructs. All assays were carried out in at least three replicates.

## 5. Conclusions

The use of an *N*-analog of the cholesterol probe to improve insertion into membrane rafts worked partially. That is, there was some raft-area selectivity, but the probe also incorporated into non-raft areas. Although the *N*-analog was also inserted into other membrane areas, it differed significantly from the *O*-version (with natural 3β-*O*-cholesterol), which did not insert into raft areas of the plasma membrane or inserted to a lesser extent. Although this is a step toward target-specific labeling of rafts and may be experimentally useful, further development is required. Attempts to preserve the ability of this probe to insert into rafts while avoiding non-raft regions of the plasma membrane are the subject of further research.

## Figures and Tables

**Table 1:** Structures of the glycan constructs. All FSLs have the same A (type 2) functional head (A2), with variations in spacers including Ad, adipoyl; Av, 5-aminovaleric acid; CMG, carboxymethylglycyl type spacer; and lipids including DOPE, 1,2-dioleoyl-*sn*-glycero-3-phosphoethanolamine; Chol, cholesterol; and *N*Chol, 3β-aminocholesterol.

No.	Designation	Structure
**1**	A2-Ad-*N*Chol	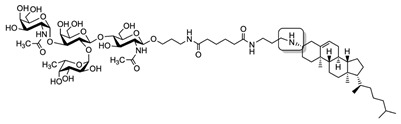
**2**	A2-Av-Chol	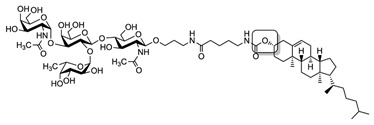
**3**	A2-Chol	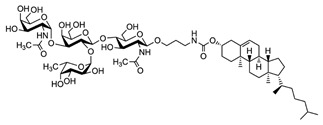
**4**	A2-CMG_2_-Av-Chol	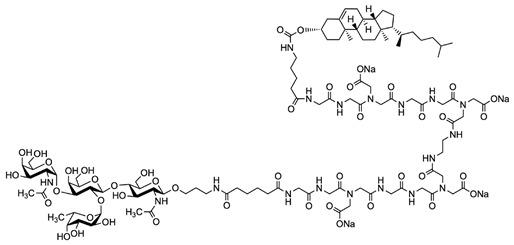
**5**	A2-Ad-DOPE	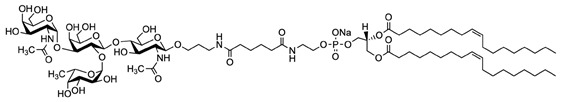

## Data Availability

The original contributions presented in this study are included in the article/Supplementary Materials. Further inquiries can be directed to the corresponding author.

## Supplementary Materials

The following supporting information can be downloaded at: https://www.mdpi.com/article/10.3390/molecules31132297/s1, Figure S1: ^1^H NMR spectrum of **A2-Ad-*N*Chol**; Figure S2: ^13^C NMR spectrum of **A2-Ad-*N*Chol**; Figure S3: HRMS (ESI) spectrum of **A2-Ad-*N*Chol**; Figure S4: EA.hy 926 cells apoptosis and viability assay before and after insertion of **A2-Ad-*N*Chol**; Figure S5: Confocal microscopy of the distribution of **A2-Av-Chol** and **A2-Ad-*N*Chol** after 5 hours incubation at 37 °C in EA.hy 926 cells.

## Abbreviations

DOPE, 1,2-dioleoyl-*sn*-glycero-3-phosphoethanolamine; DSPE, 1,2-distearoyl-*sn*-glycero-3-phosphoethanolamine; Chol, cholesterol; *N*Chol, 3β-aminocholesterol; PC-Chol, phosphatidylcholine-cholesterol; Ad, adipoyl; Av, 5-aminovaleric acid; CMG_2_, carboxymethylglycyl type spacer; EIA, enzyme immunoassay; A2, A (type 2) blood group A glycan, GalNAcα1-3(Fucα1-2)Galβ1-4GlcNAcβ; FSL, Function-Spacer-Lipid; GSL, glycosphingolipid.
