# Supplementary material for: Raft Selectivity of a Cholesterol Probe Capable of Forming an H-Bond with Phospholipids
Source: Molecules. 2026 Jul 1;31(13):2297. doi: 10.3390/molecules31132297 (PMC13362587; doi:10.3390/molecules31132297)
Supplement: Supplementary file 1 [file molecules-31-02297-s001.zip › molecules-4177301-supplementary.pdf]

Supplementary Materials

Raft Selectivity of a Cholesterol Probe Capable of Forming an H-Bond with Phospholipids

Ivan Ryzhov, Eugenia Rapoport, Polina Obukhova, Alexander Tuzikov, Mariia Sokolova, Darya Anisimova, Oxana Galanina, Sergey Khaidukov, Stephen Henry and Nicolai Bovin

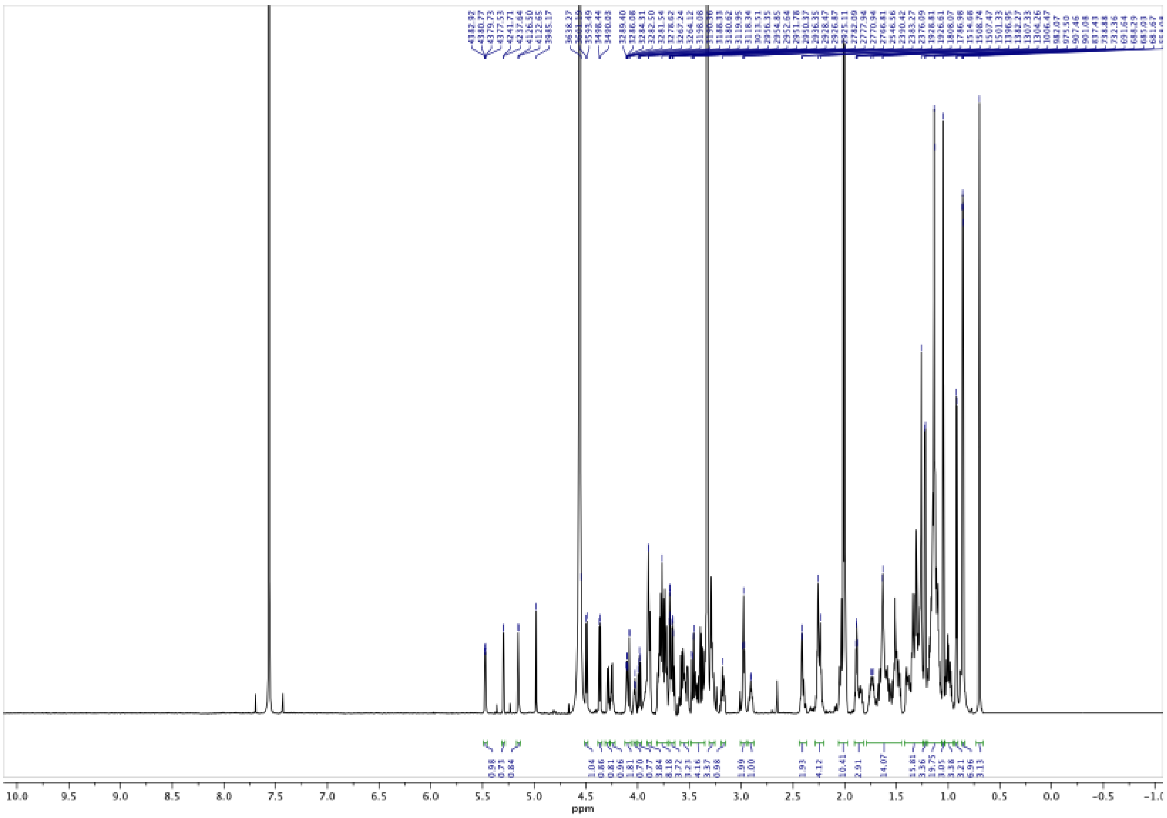

Suppl. Mat. Fig. S1. ^{1}H NMR spectrum of **A2-Ad-*N*Chol** (700 MHz, CD_3OD—$CDCl_3$, 1 : 1, 303K).

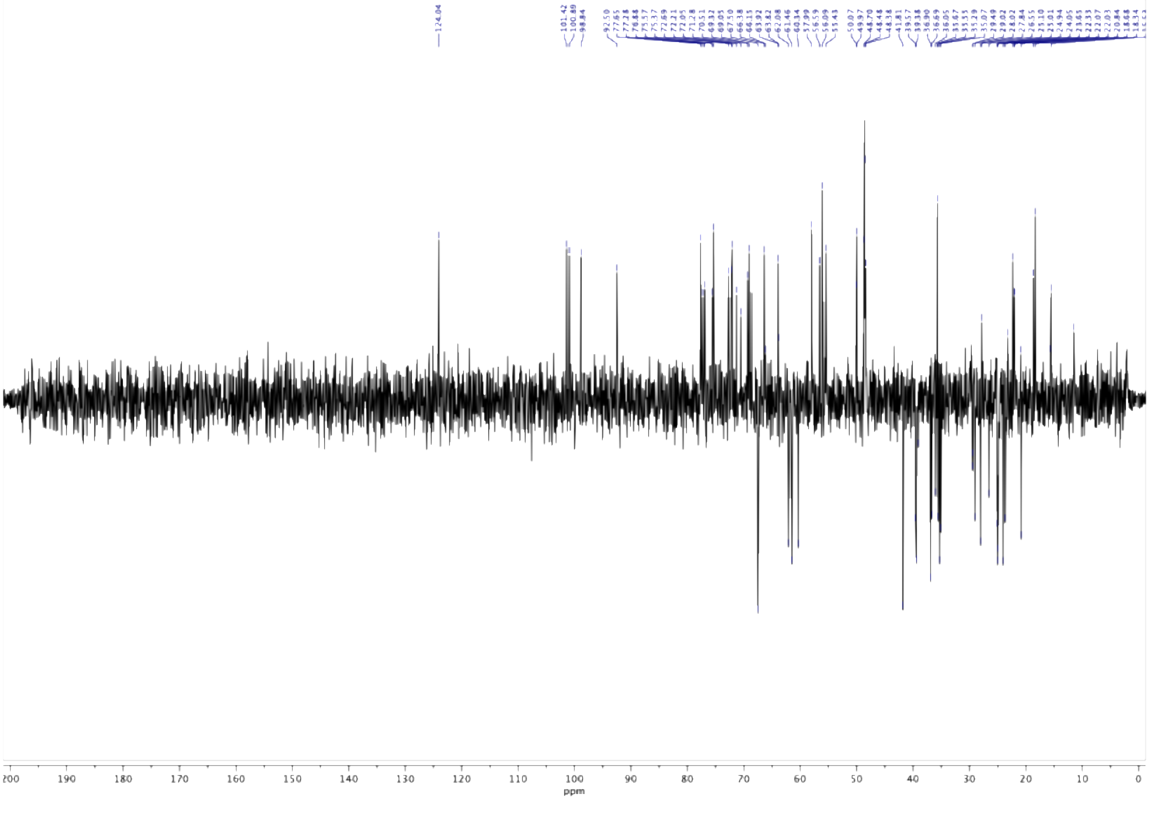

Suppl. Mat. Fig. S2. ^{13}C NMR spectrum (DEPT 135°) of **A2-Ad-*N*Chol** (201 MHz, CD_3OD—$CDCl_3$, 1 : 1, 303K).

Acquisition Parameter

Source Type	ESI	Ion Polarity	Positive	Set Nebulizer	0.5 Bar
Focus	Active			Set Dry Heater	180 °C
Scan Begin	50 m/z	Set Capillary	4500 V	Set Dry Gas	4.0 l/min
Scan End	3000 m/z	Set End Plate Offset	-500 V	Set Divert Valve	Waste

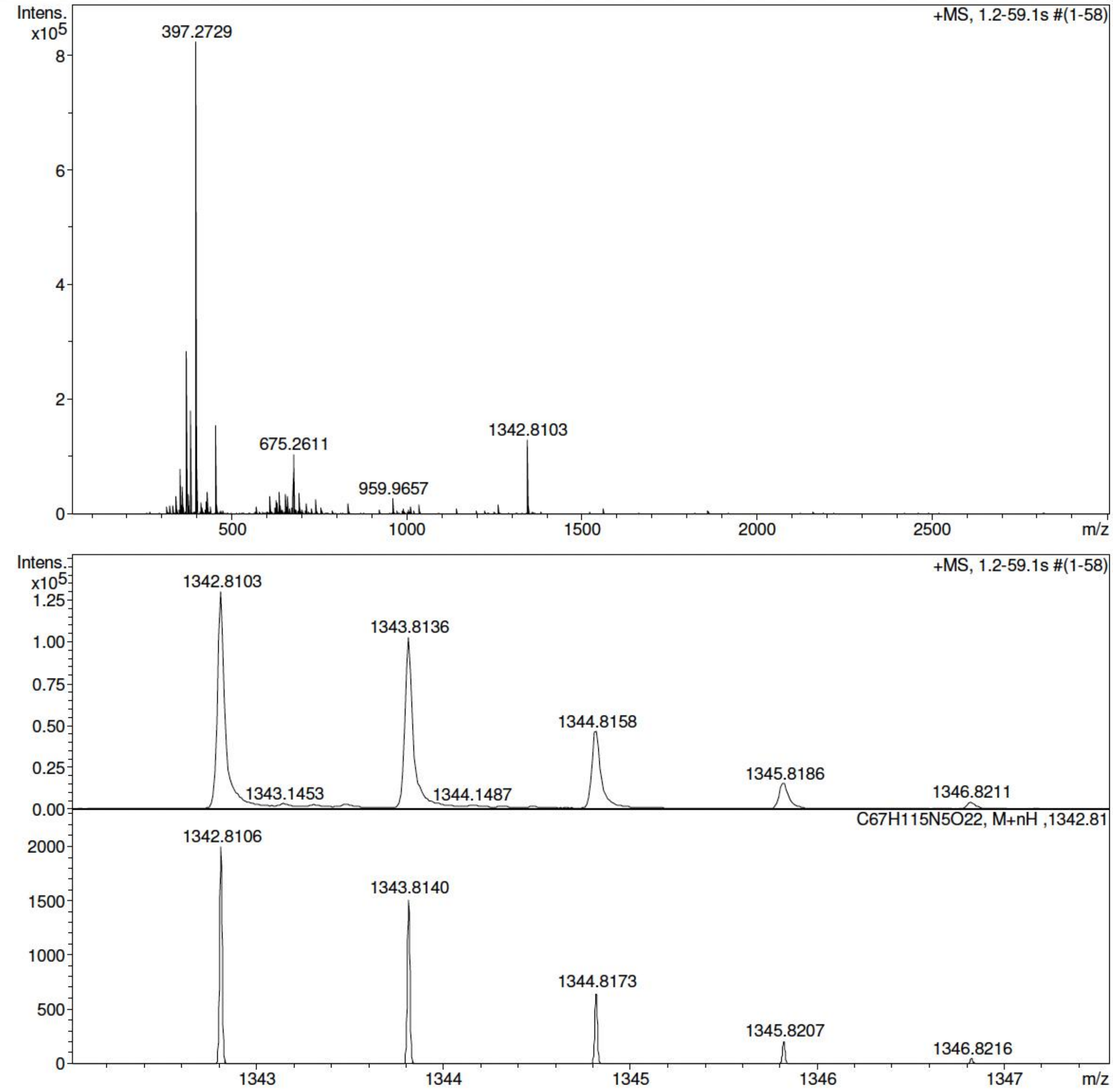

Suppl. Mat. Fig. S3. HRMS (ESI) spectrum of **A2-Ad-*N*Chol**: found *m/z* 1342.8103 $[M+H]^+$, calculated for $C_{67}H_{115}N_5O_{22}$: 1342.8106.

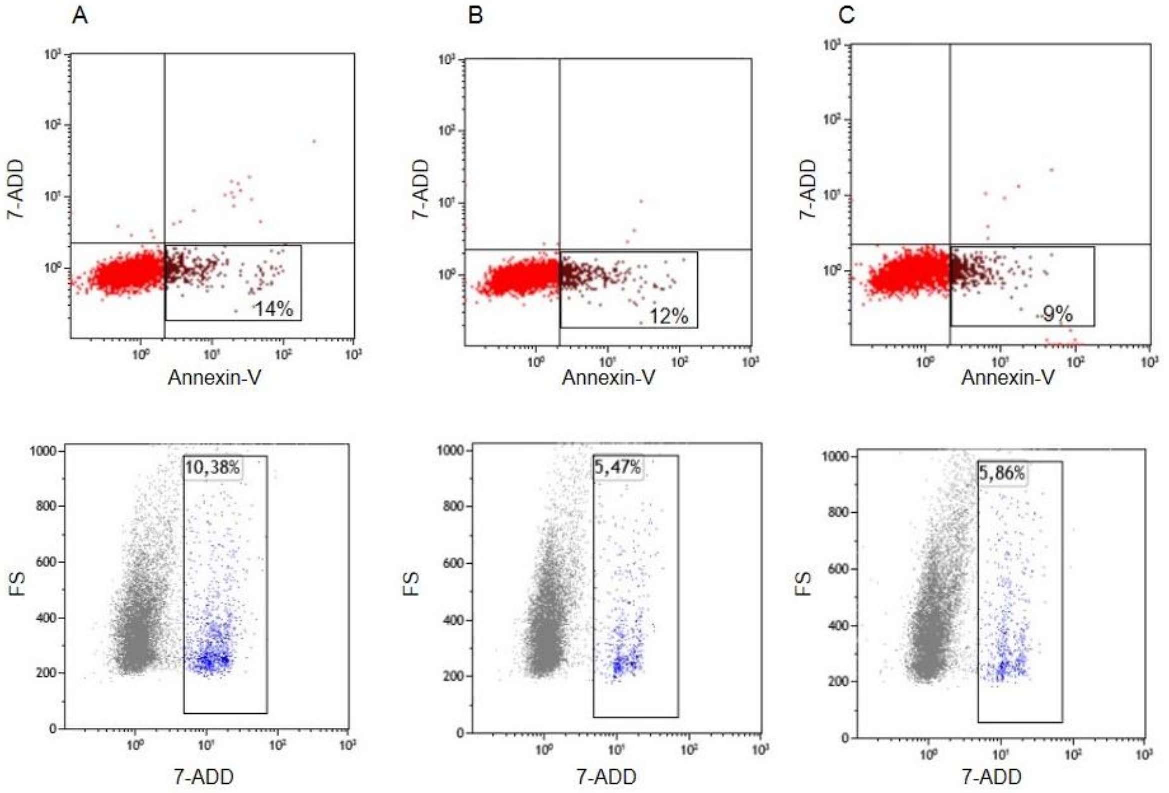

Suppl. Mat., Fig. S4. EA.hy 926 cells apoptosis (upper panel) and viability (bottom panel) assay before (A) and after insertion of **A2-Ad-*N*Chol**, 1 (B) and 5 μM (C), flow cytometry**.** In the dot-plots apoptotic cells were identified by gating logarithm of fluorescence of 7ADD vs Annexin V; necrotic cells – by gating forward scatter (FS) vs FL-7ADD, the number given in the rectangles indicate the percentage of apoptotic (upper panel) and necrotic cells (bottom panel).

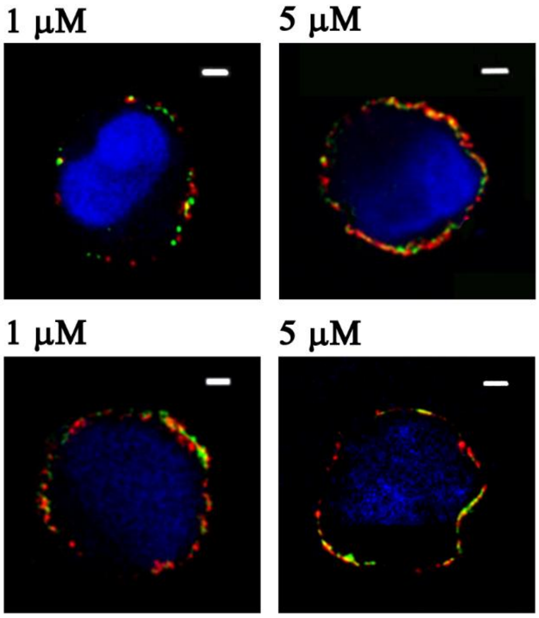

FSL	FSL entrapped in rafts relative to total FSL in the membrane, %
A2-Av-Chol, 1 μM	10
A2-Av-Chol, 5 μM	7
**A2-Ad-*N*Chol, 1 μM**	**6**
**A2-Ad-*N*Chol, 5 μM**	**8**

FSL	Area of rafts where FSL is detected, %
A2-Av-Chol, 1 μM	12
A2-Av-Chol, 5 μM	11
**A2-Ad-*N*Chol, 1 μM**	**15**
**A2-Ad-*N*Chol, 5 μM**	**15**

Suppl. Mat. Fig. S5. Confocal microscopy of the distribution of **A2-Av-Chol** (upper panel) and **A2-Ad-*N*Chol** (bottom panel) after 5 hours incubation at 37 °C in EA.hy 926 cells. Rafts were

visualized by CTB-FITC (green) while FSL constructs were stained with mouse anti-A and anti-mouse IgM+IgG Alexa Fluor (red), nuclei are stained with DAPI (blue). The white bar inset top right in each image correspond to 5 μm.
